# Lessons from the Embryonic Neural Stem Cell Niche for Neural Lineage Differentiation of Pluripotent Stem Cells

**DOI:** 10.1007/s12015-012-9381-8

**Published:** 2012-05-25

**Authors:** Valeriya Solozobova, Nicolas Wyvekens, Jan Pruszak

**Affiliations:** 1 Emmy Noether Group for Stem Cell Biology, Department of Molecular Embryology, Institute of Anatomy and Cell Biology, University of Freiburg, Albertstrasse 17, 79104 Freiburg, Germany; 2 Center for Biological Signaling Studies (BIOSS), Albertstrasse 19, 79104 Freiburg, Germany

**Keywords:** Embryonic stem cells, Induced pluripotent stem cells, Neural stem cells, Stem cell niche, Self-renewal, Differentiation, Growth factors, Extracellular matrix, Surface molecules

## Abstract

Pluripotent stem cells offer an abundant and malleable source for the generation of differentiated cells for transplantation as well as for *in vitro* screens. Patterning and differentiation protocols have been developed to generate neural progeny from human embryonic or induced pluripotent stem cells. However, continued refinement is required to enhance efficiency and to prevent the generation of unwanted cell types. We summarize and interpret insights gained from studies of embryonic neuroepithelium. A multitude of factors including soluble molecules, interactions with the extracellular matrix and neighboring cells cooperate to control neural stem cell self-renewal versus differentiation. Applying these findings and concepts to human stem cell systems *in vitro* may yield more appropriately patterned cell types for biomedical applications.

## Introduction

Pluripotent stem cells (PSCs), i.e. embryonic stem (ES) cells and induced pluripotent stem (iPS) cells represent particularly attractive cell sources for regenerative medicine [1, 2]. They exhibit vast expansion potential and tissue availability, an ability to be patterned toward the widest range of cellular phenotypes, and provide feasible options for standardization and scale-up of therapeutic cell differentiation [3, 4]. One challenge, however, remains how to control this vast potential and exploit it so to appropriately guide this cell source from pluripotency exclusively toward the phenotype of biomedical interest. Cellular heterogeneity can be considered an inherent confounding feature of PSC differentiation systems [5, 6]: in contrast to the orchestrated, highly reproducible and precisely timed development occurring during ontogeny of the embryo, a greater variety of developmental stages and cell types exist in these artificial *in vitro* development systems. *In vivo*, features of pluripotency are merely present at the earliest stages of development (blastocyst toward epiblast stage) and reliably decline after ca. five days in the mouse (Theiler stage 8) and after 10 to 15 days (Carnegie stage 5) in humans. In contrast, a remaining concern of PSC differentiation systems is the carry-over of proliferative stem cells to later stages of *in vitro* development [5, 7, 8]. In addition to contaminating pluripotent cells, the presence of precursor cells and other cell types that would never co-exist within the same developmental tissue compartment during physiological development *in vivo* may interfere with cellular patterning efforts in the dish. The key concept of such reasoning is that current *in vitro* differentiation approaches do not sufficiently take into account the interactions of cells with one another and with the resulting extracellular microenvironments in the dish. This will be of critical importance, however, as full control over proliferation and targeted differentiation of stem cells represents a prerequisite to their safe and efficient use in biomedical applications including cell transplantation and *in vitro* pharmacological screens.

We aim to exploit insights into physiological neural development to devise better *in vitro* stem cell differentiation systems for future biomedical approaches aimed at alleviating neurological disease. In the embryo, occurring at day seven in the mouse (Theiler stage 11), and ca. week four post-conception in humans (Carnegie stage 9), invaginating neural cells eventually form a tube of columnar neuroepithelial cells. Along this neural tube, a pseudostratified neuroepithelium develops that gives rise to the central nervous system (CNS), i.e. the spinal cord and brain. As the divergent macroscopic dimensions of these latter two structures demonstrate, regulation of self- renewal versus differentiation within this germinal layer must be tightly controlled: the cranial portion of the neural tube generating the rather prolific telencephalic tissue mass and the caudal portion the comparatively limited amount of neurons constituting the gray matter of the spinal cord. Insights into the mechanisms regulating the delicate balance between proliferation versus differentiation in the embryonic neuroepithelial stem cell niche will enable us to much more appropriately modulate *in vitro* conditions for the generation of specialized neural cell types from PSCs.

**Stem cell niches** are defined as microenvironments that maintain survival, self-renewal, activation, proliferation and regenerative capacity of stem cells [9, 10]. Whether in the developing embryo or *in vitro*, signaling via soluble factors, via the extracellular matrix (ECM) and direct cell-cell interactions via cell surface molecules contribute to controlling appropriate stem cell function. *In vivo*, most stem cells niches contain basement membranes and vascular elements [11]. In addition to their function as adhesion anchor points, these ensure stem cell integrity and growth control, as well as appropriate cell polarization and orientation. Furthermore, ECM components within stem cell niches are able to trap growth factors, thereby regulating their local concentration and availability.

**Neural stem cells** (NSCs) are self-renewing, multipotent cells that are present in the embryonic as well as the adult CNS. During early embryogenesis of mammals, the neural plate and neural tube consist of a single layer of proliferating neuroepithelial cells. These *primary* NSCs have the capacity to self-renew and, neurogenesis preceding gliogenesis, give rise to the neurons of the CNS and radial glia as well as to astrocytes and oligodendrocytes. These NSCs express markers including the intermediate filament nestin, and the transcription factors Pax6 and Sox2. Neuroepithelial cells extend from the ventricular (apical) to the pial (basal) surface (apico-basal polarity), and the migration of nuclei from one to another (interkinetic nuclear migration) creates the impression of a multi-layered (pseudostratified) epithelium [12]. In order to grow in numbers during early embryogenesis, neuroepithelial cells divide *symmetrically* to produce two identical daughter cells. Later, in the mouse brain after embryonic day (E)11, neuroepithelial cells switch to various modes of *asymmetric* cell divisions that generate two distinct daughter cells, a self-renewing stem cell and a differentiating neuroblast [13, 14].

During the transition to multi-layered neural tissue neuroepithelial cells produce **radial glia cells** that succeed the early neuroepithelium and exhibit many similar properties but also possess some unique glial characteristics. They express markers such as 3CB2 (a putative intermediate filament-associated protein), radial glial marker-2 (clone RC2), as well as nestin, vimentin and glial fibrillary acidic protein (GFAP), among others. Both neuroepithelial and radial glia cells are capable of self-renewal and generate neurons, intermediate progenitors (basal progenitors) and glia, and both cell types are characterized by apico-basal polarity, exhibit interkinetic nuclear migration and are nestin-positive and prominin-1-positive [13]. Radial glia also provide the substrate for migration of newly formed postmitotic neurons along their radial glial processes [15] which is critical for cortex layer formation in a defined temporal and spatial order.

While proliferation and differentiation of the nervous system of mammals is limited after conclusion of fetal development [16], certain circumscribed areas in the brain retain multipotent cells with the ability to self-renew and to differentiate into neural lineages: the subependymal layer of the subventricular zone (SVZ) of the lateral ventricles and the subgranular zone of the dentate gyrus in the hippocampus [17, 18]. Both primary fetal tissue- and adult brain-derived NSCs can be maintained and propagated *in vitro* as three-dimensional aggregates, termed neurospheres. Neurosphere formation from primary neural tissue was first explored by Reynolds and Weiss, who demonstrated the presence of expandable NSCs in the mammalian adult brain by isolating them from CNS tissue. These cells were able to generate astrocytes and neurons [19]. This technique continues to be routinely used for expansion and study of adult and embryonic NSCs.

**Fig. 1:**
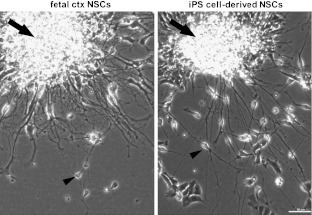
Neural stem cell proliferation and differentiation *in vitro*. Phase contrast microphotographs illustrating dense clusters of proliferative neural cells (*arrows*) which are present in NSC culture from primary cortical tissue (*left panel*), as well as in NSC cultures derived from human iPS cells (*right panel*). In both cases, differentiating, process-bearing neurons (*arrowheads*) emerge from the proliferative core. Scale bar: 50 μm

Since the derivation of human ES [1] and more recently iPS cells [2, 20], a number of neural induction protocols have been devised and optimized so to reliably and efficiently generate neuroepithelial progeny from pluripotent sources [21–23]. After induction of adherent PSC cultures with neuralizing agents such as Noggin or other bone morphogenetic protein (BMP)/Smad inhibitors [24, 25] characteristic differentiation toward neuronal, astroglial and oligodendroglial sublineages has been established. Combined with their capacity for extended self-renewal and positivity for nestin, Sox2 and Pax6, this suggests phenotypic features analogous to embryonic NSCs [23] (Fig. 1). Nevertheless, further characterization of PSC-derived NSCs is undoubtedly needed with respect to their precise developmental stage(s) and potency.

We aim to synergize and integrate what is known about the respective roles of growth factors, the ECM, and cell-cell interactions for growth and differentiation of developing NSCs. Moreover, we hypothetically discuss potential converging downstream pathways of these NSC regulators in order to exploit these insights for improved differentiation of neural cell types from PSCs.

## Microenvironmental Factors Controlling the Neural Stem Cell and Neurogenic Niche

Even free-floating neurosphere cultures arising from a single NSC after multiple cycles of divisions comprise many cells types, including NSC and various differentiated progenitors embedded within these spherical cultures [26, 27]. This underlines the ability of cells in culture to generate their own microenvironment through autocrine, paracrine and exocrine soluble signaling factors (Table 1), through secretion and modulation of ECM components (Table 2), through direct cell interactions with one another via surface molecule-mediated signaling (Table 3), as well as through mechanosensory signals related to tension gradients across the sphere [28–30]. To fully control stem cell development in *in vitro* differentiation systems we will need to better understand the influence of these processes over an NSC’s choice either to reenter the cell cycle and proliferate or to exit and differentiate.

**Table 1:** Soluble molecules and growth factors regulating NSC proliferation and differentiation in the developing embryonic nervous system

Category	Type	Notes & comments	References
Humoral/soluble factors	bFGF	promotes NSCs proliferation at early stages of neural development; promotes β1-integrin expression in neuroepithelial cells; expansion of neurospheres	Tropepe et al. 1999 [35]; Suzuki et al. 2010 [100];
	EGF	promotes NSCs proliferation, particularly at later stages of development; increases expression of β1-integrin in neuroepithelial cells; expansion of neurospheres	Tropepe et al. 1999 [35]; Lilien and Rafael 2000 [37]; Reynolds and Weiss 1992 [19]; Suzuki et al. 2010 [100];
	Cystatin-C	cooperates with bFGF signaling to induce NSC proliferation	Taupin et al. 2000 [39]
	IGF1	promotes NSC proliferation; cooperates with EGF; functional component of cerebrospinal fluid	Arsenjevic et al. 2001 [45]; Lehtinen et al. 2001 [44]
	IGF2	IGF2 mutant mice show brain development defects	Baker et al. 1997 [42]
	Insulin	promotes neural progenitor proliferation and survival; induces hypothalamic neural progenitor proliferation and astrocytic differentiation	Freund et al. 2008 [40]; Desai et al. 2011 [41]
	TGF-α	as other TGF-α members (EGF, HB-EGF) promotes NSC proliferation	Cooper and Isacson 2004 [46]
	TGF-β	tends to promote NSC differentiation; e.g., Tgf-β1 induces astrocytic differentiation of radial glia; promotes neurogenesis after stroke	Böttner et al. 2000 [48]; Aigner and Bogdahn 2008 [32]
	GDNF	pro-differentiation factor for neural progenitors; causes neurogenesis after stroke	Pahnke et al. 2004 [50]; Kobayashi et al. 2006 [49]
	BMPs	control the production of neurons, oligodendrocytes and astrocytes; decreases proliferation of NSC	Sabo et al. 2009 [51]
	NGF, NT-3, NT-4	potent regulators of neurogenesis; inhibit proliferation and promote differentiation of cortical progenitors	Huang and Reichardt 2001 [54]
	BDNF	induces premature radial glia differentiation in the developing brain; proliferating effects on embryonic NSCs even in the absence of EGF; wide role in neurogenesis	Huang and Reichardt 2001 [54]; Islam et al. 2009 [57]
	GABA	expressed by neuroblasts inhibits the proliferation of type B astrocytes in the SVZ zone of adult brain	Liu et al. 2005 [58]
	Retinoic acid	supports neural differentiation, neural patterning, axon outgrowth	Maden 2007 [33]; Siegenthaler et al. 2009 [120]
	Noggin, Chordin	neural induction via inhibition of BMP signaling	Chambers et al. 2009 [24]; Morizane et al. 2011 [25]
	MMP-2	expressed in neuroepithelia; expression decreases as maturation proceeds; promotes migration of neural progenitors	Frölichstahl-Schoeller et al. 1999 [28]; Wang et al. 2006 [65]
	TIMP-4	expressed in neuroepithelia; expression decreases as maturation proceeds	Frölichstahl-Schoeller et al. 1999 [28]

**Table 2:** Matrix components and properties involved in regulating NSC proliferation and differentiation in the developing embryonic nervous system

Category	Type	Notes & comments	References
ECM	Laminin	expressed in VZ, particularly α2 (e.g. laminin-211) and α4 chains, along the routes of migrating neurons and in the subplate; deletion of laminin α2 causes abnormalities in the composition and architecture of VZ	Loulier et al. 2009 [68]; Hunter et al. 1992 [69]
	Fibronectin	expression is observed at pial surface of VZ; during layer formation displays a speckled pattern throughout the VZ and along radial glia processes	Campos et al. 2004 [29]; Sheppard et al. 1995 [73]
	Collagen	collagen-IV is highly expressed in the VZ and the SVZ	Lathia et al. 2007 [61]; Campos et al. 2004 [29]
	Vitronectin	synergistically with Shh induces spinal motoneuron differentiation	Pons and Martí 2000 [77]
	Tenascin	distinct tenascin-R domains inhibit NSC proliferation and promote astrocytic differentiation; tenascin-C shifts NSC differentiation toward glial fate; lack of tenascin-C causes a delay of EGFR acquisition in NSC	Liao et al. 2008 [80]; Garcion et al. 2004 [82]
	Agrin	high expression in early neuronal development on the pial surface of VZ, high expression in the brain	Martin et al. 1997 [83];
	Nidogen	nidogen-1 is expressed on pial surface of VZ	Kohfeldt et al. 1998 [88]; Lathia et al. 2007 [61]
	Perlecan	expression on the pial surface of VZ; modulates FGF and Hedghog signaling in the fly	Lathia et al. 2007 [61]; Park et al. 2003 [63]
Mechanotransduction	Material stiffness	decreased stiffness promotes neuroectodermal specification and neuronal differentiation	Engler et al. 2006 [133]; Saha et al. 2008 [134]
	Tensile forces	mechanical factors modulating neural phenotype and morphogenesis	Van Essen 1997 [138]; Hayashi and Carthew 2004 [139]
	Actin	the transition from radial glia to intermediate neural progenitor cells depends on F-actin organization; actin cooperates with calmodulin in neuronal differentiation of cortical NSCs	Saffary & Xie 2011 [142]; Yu et al. 2011 [143]

**Table 3:** Surface molecules known to be expressed in the neurogenic niche of the developing embryo

Category	Type	Notes & comments	References
Surface molecules	EGFR	expression increases with development; responsive to bFGF (earlier in development) as well as to EGF (later in development)	Tropepe et al. 1999 [35]; Zhu et al. 1999 [36]; Lilien and Rafael 2000 [37]
	IGFR	widely expressed in the CNS including VZ and SVZ; mouse mutants reveal severe brain developmental defects	Arsenijevic et al. 2001 [45]; Baker et al. 1993 [42]; Beck et al. 1995 [43]
	β1-integrin (CD29)	expressed in NSCs; lower surface expression in differentiated cells; required for neuroepithelial proliferation, contributes to EGFR signaling	Campos et al. 2004 [29], 2006 [150]; Hall et al. 2006 [93]; Pruszak et al. 2009 [92]; Suzuki et al. 2010 [100]
	αv-integrin (CD51)	expressed by radial glia	McCarty et al. 2005 [102]
	α6-integrin (CD49f)	neuronal ectopia in the cortex α6-deficient brain; is expressed in NSC	Georges-Labouesse et al. 1998 [62]; Lathia et al. 2007 [61]; Loulier et al. 2009 [68]
	Ephrins	Ephrin A/EphA receptors promote neural progenitor apoptosis, leading to decrease in cortical size; loss of ephrin B leads to decrease of neural progenitors through early cell cycle exit	Depaepe et al. 2005 [103]; Qiu et al. 2008 [104]
	heat stable antigen (CD24)	low expression is observed on NSC, whereas its expression increases in neuroblasts and neurons	Calaora et al. 1996 [105]; Pruszak et al. 2009 [92]
	Dystroglycans	high expression in the developing neural tube; defects in glycosylation lead to defects in basement membranes, neuronal migration, brain morphogenesis	Sugiyama et al. 1994 [85]; Montanaro and Carbonetto 2003 [94]
	Syndecans	expression in developing neural tube; role in neuronal migration	Lathia et al. 2007 [61]; Hienola et al. 2006 [95]
	Prominin-1 (CD133)	apical area-associated protein in neuroepithelia and NSCs	Weigmann et al. 1997 [127]
	E-Cadherin (CD324)	expressed by NSCs, regulates NSC self-renewal	Karpowicz et al. 2003 [111]
	N-Cadherin (CD325)	participates in adherens junction formation; controls neuroepithelial proliferation	Lien et al. 2006 [106]; Kadowaki et al. 2007 [112]; Van Hateren et al. 2011 [115]; Chalasani and Brewster 2011 [114]
	Gap junctions, Connexins	electrochemical coupling of NE and radial glia in embryonic stem cell niche (connexins-26 and −43)	Bittman et al. 1999 [117]; Valiente et al. 2011 [116]
	FORSE1 (forebrain- surface-embryonic 1)	forebrain embryonic antigen expressed in neuroectodermal proliferating cells	Tole et al. 1995 [107]; Pruszak et al. 2007 [5]; Elkabetz et al. 2008 [21]

### Soluble Factors

There are several excellent and detailed review articles elucidating the role of growth factors, chemokines and cytokines in NSC development [31–34], and in the following we merely sketch out and exemplify major interdependent growth factor pathways contributing to controlling the neurogenic niche (see Table 1). Early in development, NSCs are mainly responsive to basic fibroblast growth factor (bFGF) signaling, with a later shift to epidermal growth factor (EGF) responsiveness [35, 36]. EGF receptor numbers increase over time of development, and while BMP signaling inhibits EGFR expression, bFGF can act as an antagonist of BMP4 and promote EGF responsiveness [37]. Empirically, mitogens such as bFGF and EGF have successfully been employed for *in vitro* expansion of embryonic as well as adult NSCs [26, 38]. The ECM-modulatory proteinase inhibitor Cystatin-C (CST3) extracted from conditioned medium of NSCs cultures has been shown to cooperate with bFGF to induce NSC proliferation [39]. In addition to extrinsic supplementation of growth factors in culture media, autocrine/paracrine factors including insulin and insulin-like growth factor (IGF) have been suggested to play a role [40, 41]. IGF-1 and −2 receptors are expressed throughout embryological CNS development, and both IGF receptors and ligands are present in the germinal zone and SVZ. *In vivo*, mouse mutants for IGF as well as IGF receptors display brain developmental defects [42, 43]. Recently, Lehtinen et al. have shown that a major functional component of the cerebrospinal fluid that regulates telencephalic NSC proliferation and, thus, brain size is the IGF-2 signaling factor [44]. IGF-1 has been shown to promote NSC proliferation, and EGF, bFGF and IGF signaling cascades intersect to regulate NSC numbers in murine neurosphere cultures *in vitro*, in that IGF-1 and EGF cooperate to promote NSC renewal, while bFGF effects appear to be IGF-1 independent [45].

In the adult subependymal zone the relevance of the transforming growth factor (TGF)-α signaling for NSC proliferation has been demonstrated by TGF-α knockout mice. Moreover, intrastriatal TGF-α infusion *in vivo* could induce proliferation of adult neural progenitor cells and recruitment to the striatum [46]. TGF-α can also promote the reversion of mature astrocytes toward earlier neural progenitors [47]. While EGF/TGF-α family members may predominantly promote NSC expansion and proliferation, the TGF-β family members may have anti-proliferative and pro-differentiating effects on NSCs and astrocytes. However, members of TGF-β family may have a beneficial effect for neuronal survival after stroke and also promote neurogenesis [32, 48]. Infusion of glial-derived neurotrophic factor (GDNF), a member of the TGF-β family, into ischemic striatum promoted neurogenesis after stroke [49]. Overexpression of GDNF, particularly together with its receptor GFRα1, causes induction of genes responsible for differentiation of neural progenitor cells [50]. BMPs control the generation of neurons, astrocytes and oligodendrocytes and decrease the proliferation of embryonic and adult neural progenitors [51]. The BMP-antagonist noggin was found to inhibit neurogenesis in neuroepithelium [52], whereas noggin expressed by ependymal cells of the adult mouse induced neurogenesis [53].

Signaling through members of the trk family of tyrosine kinase receptors, for example, neurotrophins such as nerve growth factor (NGF), brain-derived neurotrophic factor (BDNF), as well as the neurotrophin (NT)-3 and NT-4 are known as potent regulators of neurogenesis [54]. NT-3 inhibits proliferation of cortical precursors and enhances their differentiation [55]. Overexpression of BDNF in the adult rat brain enhanced olfactory and neostriatal neuronal recruitment [56]. BDNF maintains embryonic NSC proliferation through Erk, AKT and STAT3 pathways. In contrast, at the neurogenic stage, BDNF causes pre-mature differentiation of radial glia into astrocytes and glial precursors via activation of the MAPK-Erk pathway [57].

Finally, also diffusible neurotransmitters themselves can have an effect on the NSC niche: the neurotransmitter γ-aminobutyric acid (GABA) produced by neuroblasts in the adult SVZ inhibits the proliferation of type-B astrocytes, thereby creating a negative feedback loop [58]. While this paragraph exemplifies the fact that soluble factors are particularly potent modulators of NSC growth versus differentiation, mere supplementation of culture media with these factors is insufficient to fully control *in vitro* NSC development.

### ECM

Interactions of cells with the ECM regulate NSC and neural precursor behavior and their integrity within the neurogenic niche [59, 60] (see Table 2). The ventricular zone (VZ) of the embryonic brain is in contact with the pial basal lamina that is often a hallmark of a stem cell niche. Moreover, basal lamina components such as laminins, nidogen-1, perlecan, collagen IV and agrin are found throughout the VZ and SVZ, with expression patterns changing both spatially and temporally [61–63]. While the adult neurogenic zone lacks a basal lamina, it is penetrated by blood vessels and contains fractones, basal lamina-like blood vessel extensions [59, 60]. Being rich in ECM components, these zones may provide important cues for both homeostatic and regenerative neurogenesis [11, 64]. Importantly, ECM proteins can bind and release growth factors and cytokines and can themselves be proteolytically degraded [65], providing yet another mechanism of reciprocal stem cell niche regulation.

**Laminins** are large heterotrimeric glycoproteins comprised of α-, β- and γ-chains that represent a major constituent of the ECM. They play important roles in adhesion, migration, proliferation and differentiation. A number of laminin isoforms and glycoforms exist, some of which are indispensable for normal development. Others exhibit more specialized functions and their dysfunction causes defects in specific tissues and organs [66]. For example, the interaction between α6β1-, α3b1-, α6β4-integrins and the laminin α5 chain is required for development of the limbs and lungs as well as for neural tube closure [67]. Laminins are present in many tissues of the nervous system [66]. In embryonic VZ development, α2 and α4 laminins are the most prominent subtypes [61]. With respect to the embryonic neurogenic niche, laminin expression, particularly expression of laminin-211 (α2β1γ1), appears to be critical for neurogenesis in the VZ of the developing CNS [60, 61, 64]. Deletion of the α2 laminin chain caused abnormalities in the architecture and composition of the embryonic mouse VZ, underlining the relevance of interactions of laminin α2 with corresponding cell surface receptors such as integrins to provide signals necessary for NSC adhesion, morphology and growth control [68]. Furthermore, laminin is expressed as a substrate for neuronal migration, among others in the neural subplate, providing a scaffold for migrating neuroblasts [69].

**Fibronectin** is another abundant and ubiquitous glycoprotein of the ECM that affects cellular adhesion, migration, proliferation and differentiation [70]. Fibronectin-immunoreactivity appears at the blastocyst stage of mammalian embryos [71] and its deletion results in early embryonic lethality in the mouse. Heterozygous animals exhibit mesodermal defects, aberrant neural tube formation and impaired vascular development [72]. In the early pseudostratified neuroepithelia, fibronectin is seen on the pial surface of the cells [61]. Upon formation of the cortical plate, fibronectin is found at low levels in the ventricular and subventricular areas, an expression pattern which is maintained postnatally as well [29]. During cortical layer formation fibronectin is expressed by radial glia, migrating neurons and cortical neurons [73]. *In vitro*, it is commonly used as a substrate component of PSC neural differentiation protocols.

**Collagens** are a family of triple-helical proteins that provide a scaffold for the ECM and basement membrane stability. The collagen isoform α2 is present in the basal lamina of neuromuscular junctions and mediates early clustering of synaptic vesicles [74]. Other collagen isoforms, α3 and α6, participate in the maturation and maintenance of motor nerve terminals. In the VZ and SVZ as well as in the intermediate zone collagen-IV is highly present and its expression pattern changes while development proceeds, thereby implying a role in neurogenesis and migration [61]. Collagens-I and -IV are also present in fractones of the adult SVZ [59, 60].

Other components of extracellular matrix are also important for a cell’s interaction with the stem cell niche, again largely via integrin surface molecules. Martínez-Morales et al. showed that **vitronectin** is expressed in the ventral region of the neural tube and promotes differentiation of neuroepithelium to motor neurons in chick embryo *in vivo* and *in vitro* [75]. Vitronectin binds cells through integrins containing the αv-subunit. Conversely, integrins recognize the arginine-glycine-aspartate (RGD) motif on vitronectin [76]. Pons and Martí showed that sonic hedgehog can synergize with vitronectin to induce spinal motor neuron differentiation [77]. Moreover, it has been suggested that vitronectin and its receptor αvβ5-integrin are important for the elongation of parallel fibers in the cerebellum [78]. **Tenascins** are large extracellular glycoproteins. In human there are four members of the tenascin family: tenascin-C, -R, -W and -Y [79, 80]. Tenascins have also been detected in both peripheral and central nervous system. Microinjection of antibodies against tenascin-C inhibits migration of neural crest cells in the head of avian embryos [81]. Importantly, tenascin-C knockout mice display impaired NSC development which has been linked to the function of tenascin-C to promote EGFR surface expression of NSCs [82]. Also, multiple **agrin** isoforms are found in the brain neural tissue and micro-vasculature, which implies a role of the agrin molecule and its cellular receptors in the nervous system and its development. Agrin receptors include αvβ1-integrin [83], but also the neural cell adhesion molecule (NCAM, CD56) [84], α-dystroglycan [85], neuregulins [86] and others. **Nidogens** represent another glycoprotein member of basement membrane components [87], interacting with various basement membrane components such as collagen-IV and laminin. Integrins αvβ3 and α3β1 are receptors for nidogen-1, as RGD peptide as well as corresponding blocking antibodies impair cell adhesion on nidogen-1 [88]. Chondroitin sulfate proteoglycans play an important role in the fusion of neural folds upon neural tube morphogenesis [89].

### ECM-Cell Interactions

The cellular receptors for a number of neuroembryological ECM molecules including laminins, collagens, fibronectin and vitronectin comprise the integrin family of surface receptors (see Table 3). β1-integrin expression occurs early during embryonic development [90, 91] and, in a variety of tissues, β1-integrin signaling is necessary for stem cell self-renewal. Moreover, β1-integrin, the CD29 surface antigen, is highly expressed in NSCs of the VZ in the developing brain [29, 61] and marks proliferating NSCs derived from primary tissue as well as from PSCs [29, 92, 93]. In more differentiated cells types, β1-integrin expression fades and is low or absent in doublecortin-positive neuroblasts [92] and β-III-tubulin-positive cells, respectively [29]. In addition to integrins, receptors for laminins present on NSCs include dystroglycans and syndecans. They are expressed in neuron-containing regions of developing neural tube and regulate neural adhesion and migration [61, 94, 95]. Within the integrin family of surface receptors, the β1-subunit plays a central role, forming the greatest range of αβ-heterodimers. In mouse and human neurospheres β1-integrin expressing cells are observed only at the edge of the spheres, together with nestin, EGFR and laminin-α2. β-III-tubulin-positive areas exist in the center of the neurosphere, and do not express β1-integrin [29, 93]. Consistent with that, fluorescence-activated cell sorting revealed that β1-integrin high (CD29^high^) human neural cells express NSC marker mRNAs, including prominin-1 (CD133), nestin, sox2, musashi-1 and bmi-1. In contrast, β1-integrin low (CD29^low^) cells express higher levels of β-III-tubulin [92, 93]. Implication of β1-integrin in survival, self-renewal and proliferation of NSCs has been robustly established. Co-expression of the α6-subunit (CD49f) with β1-integrin has been observed in NSCs, for instance, in the VZ area *in vivo* as well as at the outer edge of neurospheres [11, 29]. Integrin heterodimers of particular functional relevance for neural development and regulation of the NSC niche include α6β1- [11, 29], α3β1- and αvβ1- heterodimers [96, 97]. Long-term blockage of ECM-cell interactions via integrins has been shown to lead to abnormalities in postnatal cortex layering [62] (reviewed in [98]). Although β1-integrin blockage in the VZ did not alter NSC differentiation, it resulted in apical detachment of radial glia cells and affected their bipolar morphology [68]. Subsequent neuronal migration and cortex development were also affected. β1-integrin-neutralizing antibodies infused to the lateral ventricle of 8-week old animals greatly decreased the number of neural precursor cells [99]. Consistent with that finding, β1-integrin knockdown in neuroepithelial cells *in vitro* has also been shown to cause a change in cell morphology, especially significant in cells grown with EGF on fibronectin-coated dishes [100]. β1-integrin loss impairs adhesion of neurospheres from postnatal mouse brain on laminin and to a lesser extent on fibronectin [101], which may reflect the possibility that fibronectin adhesion is induced by integrins present in NSCs other than β1-integrin, such as αvβ5 and αvβ8 [93, 97]. *In vitro* proliferation of neural precursors (nestin-positive, GFAP-negative) from postnatal brain on fibronectin is partly induced by β1-integrin, α5β1 or αvβ1. Laminin also induces proliferation of these cells, through β1-integrin-dependent mechanisms [29, 97].

### Cell-Cell Interactions

A broad spectrum of additional surface molecules is expressed during neural development as well as in the adult neurogenic niches and already known to be important for NSC regulation (see Table 3) [102–107]. Others remain to be studied with respect to their functional relevance. The glycan CD15 antigen (SSEA-1, Lewis-X-antigen; fucose N-acetyl lactosamine) is expressed in adult mouse neurogenic regions on type-B and -C cells of the SVZ as well is in the hippocampus and has been shown to contribute to neurogenic niche maintenance and NSC proliferation [108]. CD15 is also expressed on embryonic neuroepithelia [109], and has also been identified to label NSCs during *in vitro* neural differentiation of human PSCs [92]. Galectin-1, a lectin expressed by adult NSCs promotes proliferation of NSCs in the SVZ as well as in the hippocampal dentate gyrus in the mouse, a regulatory function requiring the interaction of galectin-1 with β1-integrin subunit on NSCs [97]. For some of these surface molecules, potent inducers have long been known, such as fucosyltransferases for expression of the CD15 epitope. Moreover, detailed insights into modulation of these glycan moieties, e.g. through sialidases have recently been gained [110]. Cadherins, a class of cell-adhesion and signaling proteins, are another group of surface molecules present on NSCs in the neurogenic zone. E-cadherin is expressed in ventricular areas of both embryonic and adult brain by NSCs and regulates self-renewal [111]. N-cadherin is necessary for the formation of adherens junctions in mouse neuroepithelial and radial glia cells and disruption of N-cadherin function causes defects in cortical organization [112, 113]. Depending on the cellular context, N-cadherin-dependent adhesion can either promote or inhibit proliferation of neural progenitors [114, 115]. Electrochemically, neural cells in the VZ interact with one another already during development by gap junctional intercellular coupling, probably via connexins-26 and −43 [116, 117]. Continued investigation of the regulatory mechanisms controlling expression and function of surface molecules that developing cells use to interact with one another and with adjacent cell populations is undoubtedly warranted. It is known that during neural induction, interactions with the underlying **mesoderm** take place, for example, by repression of BMP activity through mesodermal factors such as chordin. Analogously to that, stromal feeder cells have been exploited for neural induction of PSCs [118, 119]. Later in development, interactions with the overlaying meningeal tissues appear to be important for appropriate neuroepithelial development [120]. While insights into the precise nature of such cell-cell interactions during embryologic neuroepithelial development are still limited, we have gained considerable insight into the interaction of adult NSCs with their neighbors. For example, type B cells (NSCs) extend a process through the ependymal layer to contact the ventricle and some cells intercalate between **ependymal cells** [11, 121], and data suggests that ependymal cells modulate neurogenesis via signaling factors such as Noggin, pigment epithelium-derived factor (PEDF) and others [53, 122]. Both the SVZ as well as the hippocampal subgranular zone of adult brain are highly vascularized [11, 64]. Type B and type C (transit- amplifying precursor) cells locate closely to **blood vessels**, often contacting them, whereas neuroblasts (type A cells) locate more distal from vasculature [11, 123]. Some type B cells contact both a blood vessel and a ventricle, thereby having access to ependymal or ventricular factors (cerebrospinal fluid) and endothelial factors [11]. While this is a field of ongoing investigation, neural progenitors in the SVZ of the embryo locate and divide in close proximity to capillary branches, and embryonic vasculature in the VZ appears to be vital for the neurogenic niche and for embryonic brain development [124]. Similarly, in the adult brain cell adhesion to blood vessels in SVZ appears to be β1-integrin-dependent. Infusion of the α6-antibody GoH3 to the lateral ventricle of mouse brain causes detachment of NSC from the vascular surface [11], and association with blood vessels can be enhanced by activating quiescent NSCs toward cell cycle reentry, which is correlated with increased β1-integrin expression [64]. Neuroblasts (neuronal precursors or type A cells) migrating tangentially via the rostral migratory stream to the olfactory bulbs to form interneurons are ensheathed by **astroglia** [125], secreting factors that are likely to promote the process of migration. Thus, cellular interactions with neural progeny from the stem cells themselves and other immediate neighbors such as vascular, ependymal cells and glial cells play an essential role in maintaining the critical balance of proliferation versus differentiation in NSC niches.

### Supracellular Context, Cellular Orientation and Polarity

For *in vitro* differentiation of PSCs, we routinely apply complex combinations of growth and patterning factors and supply appropriate recombinant or synthetic substrates modeled after embryonic ECMs in a sequence and time frame largely equivalent to embryological development. What is lacking is a detailed understanding of how cell-cell communication and interdependent structural organization (cell and tissue morphogenesis) contribute to NSC development *in vivo* as well as *in vitro*. The above humoral factors, ECM components and surface molecules enable a cell to sense (and modulate its interaction with) its immediate environment, and its position and proper orientation within it. Extending between the basal and pial surfaces of the germinal layer, embryonic neuroepithelia display a polarized character, an asymmetric distribution of cytoplasmic as well as membrane-bound structures and organelles (apico-basal polarity). For instance, cadherin-associated adherens junction complexes, tend to localize toward the apical surface [126]. On the surface of neuroepithelial cells, apical area-associated proteins including prominin-1 [127], cdc42 and PAR3/PAR6/aPKC complexes have been identified [13]. As previously indicated, the apico-lateral membrane of neuroepithelial cells is also rich in α6β1-integrin, and localization of β1-integrin complexes has been observed apico-laterally, associated with adherent junctions [61, 68]. Such asymmetrical β1-integrin distribution on the membrane enables the unequal inheritance by daughter cells [61]. Importantly, the apico-basal polarity of neuroepithelial cells and radial glia forms the basis for the switch between asymmetrical and symmetrical cellular divisions [126]. Vertical cleavage planes bisecting the apical surface result in symmetric cell division. Horizontal cleavage causes unequal partitioning of cellular components and thereby asymmetric inheritance [128, 129]. As unequal distribution of β1-integrin between daughter cells has been observed, β1-integrin might be spread unequally in daughter NSC and progenitor cell depending on mitotic spindle orientation and its own membrane distribution [61]. Blocking the interaction of the ECM with β1-integrin alters NSC cleavage plane orientation in the embryonic brain, and fewer divisions were observed with horizontal cleavage planes [68]. Whether this is caused by cell detachment from the apical surface of the lateral ventricle or absence of β1-integrin signaling itself has not been entirely clarified. Detachment from the neurogenic niche is one consequence of diminishing the ECM-integrin connection [11, 64, 68]. Loss of contact may change the position of the NSCs or neural precursors, thereby leading to differential exposures of daughter cells to various extracellular factors. Laminin-γ1 mutant zebrafish reveal abberant interkinetic nuclear migration, basal dislocation of mitotic nuclei in neuroepithelia and randomized cleavage planes mediated through focal adhesion kinase (FAK) activation, proposing a putative role of integrins in neuroepithelial cytokinesis [130]. Potentially, integrins regulate mitotic spindle organization in mammalian neuroepithelia, as β1-integrin is known to regulate mitotic spindle assembly and cytokinesis in Chinese hamster ovary (CHO) cells [131] and regulation of spindle orientation by integrin-mediated adhesion in epithelial cells and in HELA cells has been shown [132].

Thus, as in the embryo, neural stem and progenitor cells undergoing differentiation in the dish base their fate decisions on the context of appropriate growth factor concentrations, ECM interactions and on cell-cell signaling. Furthermore, **cell density** is a factor empirically taken into consideration but still rather poorly understood. In a now classic paper by Tropepe et al., 1999, an influence of cell density on the generation of primary tissue-derived NSCs was observed: EGF-mediated mitogenic effects were more profound under high density plating conditions (as opposed to bFGF-mediated mitogenic effects) [35]. The influence of cell-cell interactions on PSC neural differentiation pathways was recently implied empirically, as high density cultures of PSCs undergoing neural differentiation tend to generate CNS-neuronal derivatives, while lower density conditions seem to favor neural crest development [24]. At the stage of pluripotency, **substrate stiffness** has a profound effect on cell fate decision; enhanced matrix stiffness resulting in mesodermal offspring, while decreasing stiffness has been shown to promote neuroectodermal lineage specification [133]. At more committed stages, NSCs show a propensity toward neuronal differentiation on softer substrates (100–500 Pa), while glial differentiation is promoted at higher stiffness conditions (e.g., 10,000 Pa) [134]. Hypothetical pathways involved in sensing the extracellular conditions with respect to cell density could include signaling mechanisms related to contact inhibition and growth control (Hippo pathway) [135–137] and integrin signaling, respectively. Tensile forces and ECM-integrin-mediated anchoring can lead to direct activation of integrin pathways via FAK, Shc and other downstream signaling cascades [116, 138, 139]. In addition, via direct linkage to cytoskeletal components integrin and cadherin signaling can also lead to modulation of a variety of actin-regulated and other mechanotransductory pathways [140–143].

## Integration of the Signaling Pathways

In the previous sections, we have illustrated the critical relevance of growth factor signaling, of ECM molecules, the integrin family of cell receptors and other surface molecules as well as recently identified mechanisms of polarity, mechanotransduction and cell density-dependent signaling for the phenomena of NSC growth control and differentiation. How do these extrinsic factors get translated to cell fate decisions? What are the underlying intracellular signaling mechanisms and how could they be integrated for appropriate control of growth versus differentiation?

**Fig. 2:**
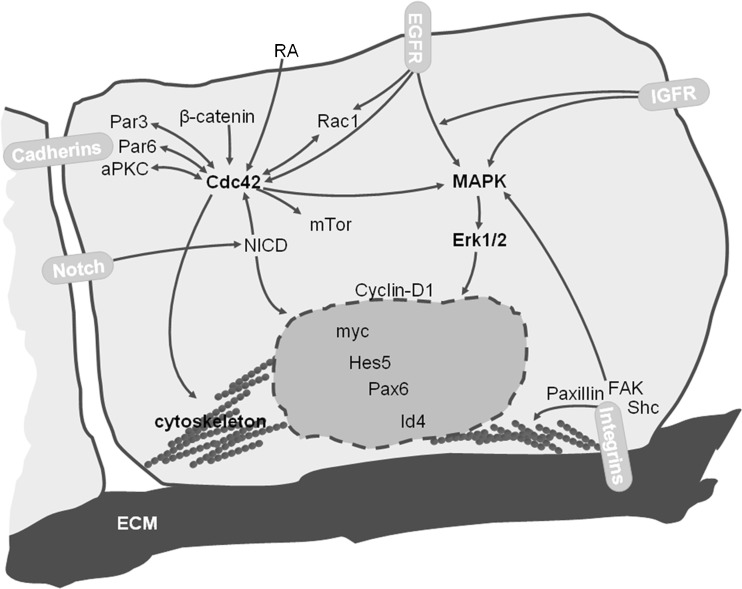
Schematic exemplifying major pathways and signaling components involved in embryonic NSC proliferation and differentiation. Cell-matrix and cell-cell interactions cooperate with tissue gradients of diffusible molecules and secreted factors from the CSF. Cdc42 and MAPK-Erk signals represent key pathways able to integrate growth factor-derived signaling (EGF, bFGF, IGFs) with adhesion and mechanotransductory signaling mediated by cadherins and integrins. Nuclear downstream factors such as myc, Hes5, Pax6, Id4 exemplify transcriptional regulators of NSC self-renewal

It is sensible to hone in on pathways that are differentially affected by the interplay of the above components of the NSC niche (see Fig. 2; Table 4). For example, bFGF and EGF are both able to increase β1-integrin expression in mouse neuroepithelial cells [100], yet bFGF has a more prominent effect on β1-integrin gene expression than EGF, whereas stimulation by EGF increases cell surface localization of β1-integrin. Treatment with **MAPK pathway** inhibitors decreases the level of β1-integrin in neuroepithelial cells grown in bFGF or EGF in a dose-dependent manner, underlining the involvement of MAPK pathway signaling in EGF- or bFGF-stimulation of β1-integrin expression [100, 144]. Similarly, β1-integrin expression in the neurospheres from postnatal mouse brain is higher in the presence of EGF than of bFGF. Interestingly, bFGF, in contrast to EGF, strongly induces EGFR expression in neurospheres [29, 101]. Cells expressing high levels of β1-integrin form more neurospheres and this effect is more prominent for EGF-grown neurospheres than for bFGF-grown neurospheres [29, 93]. Data obtained suggest that proliferation control by β1-integrin occurs through regulation of MAPK signaling, since genetic deletion or antibody blocking of β1-integrin in neurospheres grown in the presence of bFGF and EGF leads to a decrease in MAPK activation and cell proliferation [29, 100]. Subsequent passaging of neurospheres restores normal level of MAPK activation, which implies the presence of alternative pathways to substitute the lack of β1-integrin [29]. Similarly, *in vitro* knockdown of β1-integrin in mouse neuroepithelial cells also results in decreased proliferation in the presence of bFGF or EGF [100]. However, deletion of β1-integrin in conditional knockout experiments did not reveal abnormalities in the VZ [145]. Nevertheless, presence of laminins and β1-integrin in the VZ of developing mouse brain [29, 61] may suggest that interaction between them is important for NSCs and long-term absence of them could be compensated. EGF- and bFGF-driven neurospheres isolated from rat postnatal brain with β1-integrin deletion are smaller in size compared to wild-type neurospheres, while the amount of formed neurospheres is not affected by lack of β1-integrin [101]. Mutant neurospheres have a lower percentage of nestin-positive cells and a higher percentage GFAP-positive and β-III-tubulin-positive cells. Both increased apoptosis and reduced proliferation of nestin-positive progenitor cells were observed in β1-integrin-deficient neurospheres. The authors demonstrated that β1-deficiency sensitizes neurospheres to the lack of growth factors EGF and bFGF, although maintenance of progenitors was not affected by the absence of β1-integrin when cells were grown together with EGF and bFGF [101]. It has been shown as well that **Erk 1/2** activation promotes proliferation and inhibits neuronal differentiation of NSC [146, 147] and Erk is known to be a molecule that can be affected by β1-integrin signaling, though integrins are not the only activators of the Erk pathway. Thus, an important triad of integratory signals appears to be the balance of EGF and bFGF signals with β1-integrin and downstream MAPK signaling as one potential converging signaling pathway.

**Table 4:** Signaling pathways and signaling molecules involved in regulating growth and differentiation in the embryonic NSC niche

Category	Type	Notes & comments	References
Signaling pathway components	MAPK/Erk	proliferation of NSC; neural specification of ES cells requires Erk1/2 activation (while p38-MAPK inhibits NSC proliferation)	Campos et al. 2004 [29], 2006 [150]; Stavridis et al. 2007 [147]; Learish et al. 2000 [144]
	Notch	deficiency in components of notch signaling pathway (Hes1, Notch-1, RBP-Ik, PS1) decreases NSC number; required for NSC proliferation, regulates neuronal versus glial fate specification	Nakamura et al. 2000 [149]; Hitoshi et al. 2002 [148];
	FAK	interkinetic nuclear migration and planar division of neuroepithelia; adhesion during radial glial-mediated neuronal migration	Tsuda et al. 2010 [130]; Valiente et al. 2011 [116]
	Rac1	deficiency in rac1 causes reduction of neural progenitors and microcephaly in mice	Chen et al. 2008 [153]
	Cdc42	maintenance of apical-basal polarity and NSC self-renewal	Cappello et al. 2006 [154]
	Hippo	Yap1/TEAD (downstream targets of Hippo/Mst1/2) regulate neural progenitor numbers in the chick	Cao et al. 2008 [136]
	mTOR	as a potential target of cdc42 might induce NSC proliferation	Magri et al. 2011 [155]

**Notch** signaling represents another important signaling pathway for stem cell niche regulation, including in the nervous system, controlling NSC maintenance as well as neuronal/glial fate decisions [148, 149]. β1-integrin-dependent control of NSC proliferation is partially also mediated by Notch signaling [150]. β1-integrin and Notch are both co-expressed in VZ and in neurospheres. β1-integrin interacts with the Notch intracellular domain (NICD) and may modulate NICD nuclear/membrane distribution depending on the active/inactive state of β1-integrin. Coordination of β1-integrin and EGFR is required for NICD internalization from caveolae-positive lipid raft domains to non-raft domains in mouse neurospheres and ES cells-derived NSCs. Loss of β1-integrin results in decreased Notch processing and prevents its downstream proliferative activities, thereby decreasing neurosphere formation. Since Notch is required for the successive generation of neurons and glia, the study of Campos and colleagues reveals a potential way of β1-integrin control in radial glial/neuronal differentiation of NSC through the modulation of Notch signaling [150]. PEDF secreted by ependymal cells and endothelial structures in the adult SVZ has been shown to maintain self-renewal and multipotency of NSCs by increasing expression of the Notch pathway effectors Hes-1 and −5 as well as of Sox2 [122].

Members of the Rho family of guanosine triphosphate (GTP)ases are additional players in control of neural progenitors. They interpret an array of upstream signals including those emanating from integrin activation [151], and regulate the cytoskeleton, proliferation and apoptosis, interacting with and integrating mechanosensory and morphogenetic pathways [152]. In embryonic mammalian neural progenitors, Rac1-deficiency can cause apoptosis and cell cycle exit, resulting in diminishing NSC pools [153]. Moreover, the closely associated GTPase **cdc42** controls NSC renewal in the developing forebrain [154]. Deletion of cdc42 accompanied by loss of apical complex and adherens junctions results in loss of self-renewal, i.e. of maintenance of an NSC, and enhanced generation of neuroblasts. Eventually, the NSC pool is prematurely depleted in cdc42 knockout mice. Conversely, stimulating cdc42 signaling may help to recruit NSCs from adult neurogenic areas. Upstream of cdc42, links to the aforementioned cadherin and integrin molecules are clear, as cdc42 is linked to adhesion as well as to polarity. Other upstream factors include retinoic acid, as well as delta/Notch signaling, and links to mammalian target of rapamycin (**mTOR**) signaling have also been recently suggested [155]. In P19 multipotent embryonic carcinoma cells, Endo et al. have shown that cdc42 can induce Hes-5 and Pax6 gene expression [156], and basic helix-loop-helix (bHLH) family members are powerful regulators of NSC proliferation versus differentiation on a transcriptional level [157]. Hes-1 and Hes-5, are required for NSC self-renewal [158]. Pax6, highly expressed in neurogenic niches, is a multifunctional transcription factor, essential for both embryonic and adult neurogenesis. It is involved in neural tube patterning, neuronal migration and formation of neural circuits [159]. Correspondingly, the group of inhibitors of DNA binding (Id) proteins has been implicated in NSC regulation as dominant-negative regulators of bHLH factors [160, 161]. For example, Id4−/− mice show a smaller telencephalon [162]. Interestingly, Id proteins, in turn, seem to be able to regulate adherence to the neural stem cell niche by maintaining high levels of RAP1 as a positive modulator of integrins [163]. In addition to such anchoring and to intercellular signals, the decision of NSCs whether to proliferate or differentiate ultimately converges on the level of **cell cycle** regulation. The Myc family of transcription factors, potent cell cycle regulators, are also involved in fundamental developmental processes in the nervous system [164, 165]. Myc is essential for the rapid expansion of neural progenitors and regulates differentiation. Via modulation of cell cycle components such as cyclin D1, p16Ink4a or p21CIP1 myc (and also Id proteins) are thought to execute an inhibitory function on differentiation [166]. There is a line of evidence suggesting that lengthening the cell cycle without arrest triggers differentiation [167, 168]. β1-integrin and EGFR are both required for full activation of MAPK signaling and proliferation and can influence the cell cycle, and asymmetric surface distribution of EGFR and resulting divergence of cell fates has been demonstrated [169]. Since β1-integrin is able to synergize with EGFR and Notch signaling to induce proliferation [29, 100, 150], unequal partitioning of these and other receptors or signaling components may differentially affect cell cycle progression and differentiation of the generated neural progeny.

## Conclusions and Summary

The embryonic neurogenic niche is represented by a complex and well-orchestrated network of intercellular cues such as growth and other soluble factors, ECM components and surface molecules which jointly control NSC renewal and fate specification. By taking advantage of human PSC *in vitro* differentiation, fundamental mechanisms of basic human neuroembryology and NSC regulation can for the first time be studied and modulated in accessible and controllable experimental systems. Combined with insights from rodent studies, this may eventually contribute to enabling future *in vivo* recruitment of neurons from the human NSC niche in the adult [46]. In the immediate future, efforts that take into account all parameters guiding cellular decisions in the NSC niche will likely yield more appropriate neural differentiation protocols from human PSCs. Given the extended duration of the differentiation protocols (weeks to months), fine-tuning the concentrations and time frame of combinatorial exposure to growth and patterning factors will remain essential [6, 24]. In addition, beyond commonly applied purified ECM substrates, a range of recombinant or novel synthetic substrates will further enhance NSC culture specificity and standardization [10, 170]. Micro-/nano-patterned surfaces or 3D scaffolds modified to provide bioactive peptides for anchoring, options for electrochemical modulation and mechanical cues will contribute to mimicking the physical aspects of the physiological NSC niche [134, 141, 170]. Extended characterization of specific neural surface marker signatures will not only be exploited to select and isolate specific subsets of biomedical interest, but will also contribute to enhancing our understanding of the functional relevance of these molecules for intercellular signaling [21, 92]. While still far from understanding the intricacies of neural morphogenesis, more organotypic complex 3D PSC differentiation systems have already successfully been applied [171]. Taken together, regardless of the remaining challenges, the convergence of efforts ranging from fundamental cell biology to bioengineering holds great promise for the generation of more physiologically patterned neural cell types from human stem cells.
